# Right Coronary Artery Dominance in Cadaveric Human Hearts in Department of Anatomy of a Medical College: A Descriptive Cross-sectional Study

**DOI:** 10.31729/jnma.7432

**Published:** 2022-04-30

**Authors:** Nripendra Tiwari, Deepesh Budhathoki

**Affiliations:** 1 Department of Anatomy, Kathmandu Medical College and Teaching Hospital, Duwakot, Bhaktapur, Nepal

**Keywords:** *cardiac*, *coronary arteries*, *dominance*

## Abstract

**Introduction:**

Cardiac coronary dominance plays a significant role in different clinical conditions and diseases of the heart. As the people of developing and developed nations are having global coronary artery diseases, it is mandatory to have knowledge of coronary artery diseases including cardiac coronary dominance. The aim of this study is to find out the prevalence of the right coronary artery dominance in cadaveric human hearts in a medical college.

**Methods:**

A descriptive cross-sectional study was conducted among all 52 preserved hearts as well as the heart isolated from cadavers obtained from the teaching hospital. The study was conducted from 24^th^ June, 2020 to 24^th^ December, 2020 after obtaining ethical clearance from the Institutional Review Committee (Reference number: 2306202004). All cadaveric heart specimens were laelled with numbers and photographed for easy description of anatomical variation related to the coronary artery. Data were entered into Microsoft Excel 2007 and analysed using Statistical Package for the Social Sciences version 20.0. Point estimate at 90% Confidence Interval was calculated along with frequency and proportion for binary data.

**Results:**

Out of 52 undissected isolated cadaveric hearts, the right cardiac dominance was found in 42 (80.76%) (71.77-89.75 at 90% Confidence Interval). The mean diameter of the right coronary artery was found to be 4.06±0.55 mm.

**Conclusions:**

The prevalence of right cardiac dominance in isolated cadaveric hearts was similar to the studies done in a similar setting.

## INTRODUCTION

The major blood supply of the heart comed from the right and left coronary arteries. Cardiac dominance is based on the artery that provides the posterior interventricular branch supplying the back of the interventricular septum. As early as 1938, Schlesinger considered the cardiac dominance supplying the posterior interventricular sulcus.^1^ The global incidence of sudden cardiac death ranges from four to five million cases per year.^2^

Cardiac dominance plays an important role in different pathologies of the heart.^3^ Diseases of the coronary artery are quite common globally as well as in Nepal. It is the most common cause of death among non-communicable diseases in Nepal.^4^ More than 70% of sudden cardiac deaths are because of coronary artery diseases.^5^ The knowledge of the coronary arteries is important for the improvement of medical and surgical facilities for patients suffering from coronary artery diseases.^6^

The aim of this study is to find out the prevalence of the right coronary artery dominance in cadaveric human hearts in a medical college.

## METHODS

A descriptive cross-sectional study was conducted in the Department of Anatomy from 24^th^ June, 2020 to 24^th^ December, 2020 after obtaining ethical clearance from the Institutional Review Committee (Reference number: 2306202004), Kathmandu Medical College and Teaching Hospital, Duwakot, Bhaktapur, Nepal. All the preserved hearts, as well as obtained hearts obtained from cadavers, were included in the study whereas the decayed hearts and hearts with torn vessels were excluded from the study. A convenience sampling technique was used and sample size calculated according to the following formula:

n = (Z^2^ × p × q) / e^2^

  = (1.645^2^ × 0.79 × 0.21) / 0.1^2^

  = 45

Where,

n = minimum required sample size
Z = 1.645 at 90% Confidence Interval (CI)
p = prevalence of right coronary artery dominance, 79%^7^
q = 1-p
e = margin of error, 10%

The minimum calculated sample size was 45. However, a sample size of 52 was taken. All the isolated cadaveric hearts were included in the study irrespective of their age, sex and weight. The coronary arteries were traced through the epicardium and subepicardial adipose tissue. The epicardium layer of the heart was removed and coronary arteries were exposed, performing gross and microdissection for determining the dominance of the heart. Dissection was performed using instruments like pointed, blunt and toothed forceps, scalpels and straight as well as curved scissors. The dissection of cadaveric hearts was conducted in free time without hampering the teaching and learning activities at the Department of Anatomy. When the posterior interventricular artery (PIVA) was a branch of the right coronary artery, it was termed right cardiac dominance and when PIVA was a branch of the left coronary artery, it was left cardiac dominance. The balanced or codominance pattern was defined as PIVA having supply from both right and left coronary arteries. The external diameter at the origination of the right and left coronary arteries were measured using 0.01 mm sensitive digital callipers. Data was collected by a selfdesigned questionnaire in a written form to obtain the necessary information on the variables of the study. The number of the preserved and dissected hearts obtained from cadavers were labelled, dissected and observed in detail, photographed for easy description. Data collected was compiled in Microsoft Excel 2007 and further analysed by Statistical Package for the Social Sciences version 20.0. Point estimate at 90% Confidence Interval was calculated along with frequency and proportion for binary data.

## RESULTS

**Figure 1:**
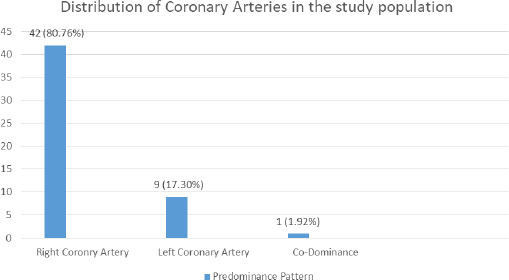
Coronary artery dominance among cadavers (n= 52).

Out of 52 undissected cadavers, the right cardiac dominance was found in 42 (80.76%) (71.77-89.75 at 90% Confidence Interval) and left cardiac dominance was found in 9 (17.30%) cadaveric hearts. The balanced or codominance pattern having PIVA supply from both right and left coronary arteries was found in only 1 (1.92%) of cadaveric hearts (Figure 1).

The mean external diameter of the left coronary artery at its origination site was found to be 4.74±0.66 mm and that of the right coronary artery was 4.06±0.55 mm (Table 1).

**Table 1:** External diameter (in mm) of right coronary artery and left coronary artery (n= 52).

Arteries	Mean±Standard Deviation
Right coronary artery	4.06±0.55
Left coronary artery	4.74±0.60

## DISCUSSION

The results of the present research showed that the right cardiac dominance was prevalent in 42 (80.76%), left cardiac dominance in nine (17.30%) and one (1.92%) of human hearts among 52 cadaveric sample representatives obtained from the Department of Anatomy at Tertiary Teaching Hospital. A similar result was found with 79% of predominant right cardiac dominance.^7^ Similar results with a slight difference i.e. 83.3% of the studied population with right cardiac dominance and 13.3% with left cardiac dominance.^6^ These results are not consistent with one of the study done, who found 70% of right cardiac dominance, 18.53% of left cardiac dominance and 11.43% of codominance.^8^ The cause for the difference could be due to differences in the sample size, age, and sex of the study population. One of the study done showed only 62.5% of right cardiac dominance and 12% of left cardiac dominance.^9^ The likely cause could be due to research performed in angiographies of living subjects rather than in cadavers as done in the present study. Similarly, we researched the mean external diameter of the left coronary artery was found to be 4.74±0.60 mm and that of the right coronary artery was 4.06±0.55 mm. This finding was comparable to another study which reported the mean external diameter for the left coronary artery as 4.34±2.01 mm.^10^ According to the research carried out in a similar setting on coronary variations, found left coronary artery had an average outer diameter of 4.44±1.79 mm and the right coronary artery with an average diameter of only 3.32±0.79 mm. ^11^ The current study is not inconsistent with an average diameter of a right coronary artery. The cause for the difference could be due to differences in the age, sex, humidity of the research place and embalming techniques and preservative methods of cadaveric specimens.

## CONCLUSIONS

The prevalence of right cardiac dominance in isolated cadaveric hearts was similar to the studies done in a similar setting. The finding can be important for clinical and surgical interventions.
